# HIF-2α downregulation in the absence of functional VHL is not sufficient for renal cell differentiation

**DOI:** 10.1186/1475-2867-7-13

**Published:** 2007-06-28

**Authors:** Michael D Hughes, Erilda Kapllani, Ashlynn E Alexander, Robert D Burk, Alan R Schoenfeld

**Affiliations:** 1 Department of Biology, Adelphi University, Garden City, NY 11530-0701, USA; 2 Departments of Microbiology and Immunology, Pediatrics, and Epidemiology and Social Medicine, Marion Bessin Liver Research Center and Albert Einstein Cancer Center, Albert Einstein College of Medicine, Bronx, New York, USA

## Abstract

**Background:**

Mutational inactivation of the von Hippel-Lindau (VHL) tumor suppressor gene has been linked to hereditary as well as sporadic clear cell renal carcinomas. The product of the VHL gene, pVHL, acts to target hypoxia-inducible factor alpha (HIF-α) subunits for ubiquitination and subsequent degradation. Using an RNA interference approach to lower levels of HIF-2α in two different renal cell lines that lack functional pVHL, we have tested the contribution of HIF-2α toward cellular pVHL activities.

**Results:**

Knockdown of HIF-2α resulted in cell cycle arrest of renal cells that were grown on collagen I, indicating that this pVHL function is dependent on HIF-2α regulation. However, cellular morphological changes and downregulation of integrins α5 and β1, which were seen upon pVHL replacement, were not faithfully phenocopied by HIF-2α reduction. Moreover, fibronectin deposition and expression of renal cell differentiation markers were observed in cells containing replaced pVHL, but not in HIF-2α knockdown cells, indicating that these pVHL functions may occur independently of HIF-2α downregulation.

**Conclusion:**

These results indicate that HIF-2α regulation is not sufficient for pVHL-induced renal cell differentiation. We hypothesize that in addition to HIF-2α dysregulation, abrogation of additional pVHL functions is required for the initiation of renal carcinogenesis.

## Background

von Hippel-Lindau (VHL) disease arises from heterozygous germline mutations in the VHL tumor suppressor gene, which resides on chromosome 3p25, and is characterized by clear cell renal carcinomas, hemangiomas, pheochromocytomas, as well as other tumor types [1,2]. The development of tumors in VHL disease results from loss or inactivation of the remaining wild type allele, leading to an absence of functional VHL protein [1]. Somatic VHL mutations are also common in sporadic clear cell renal carcinoma and hemangioblastomas (reviewed in [2]). Restoration of VHL function is sufficient to suppress *in vivo *tumor formation in VHL-defective renal carcinoma cells [3,4].

The VHL gene produces two protein products due to an internal translation initiation start site at codon 54 [3,5,6]. The larger protein produced from the normal start site is a 213 amino acid protein approximately 24–30 kDa, (VHLp30), and the shorter produced from the internal start site is an 18–19 kDa isoform (VHLp19) of 160 amino acids. The shorter form has been shown to contain full tumor suppressor function [3,5,6]. Both protein products of the VHL gene (collectively called pVHL) contain an alpha and beta domain [7]. The alpha domain associates with elongin B, elongin C, Cul2 and Rbx1 and this pVHL complex acts as a ubiquitin E3 ligase [8-13]. HIF-α, the alpha subunit of the heterodimeric transcription factor hypoxia inducible factor (HIF), binds to pVHL's beta domain and is the best-known substrate of the pVHL E3 ligase complex [14,15].

One of the conditions for pVHL to successfully bind to the HIF-α subunit is the presence of oxygen [16]. The HIF-α subunit has an oxygen dependent domain that contains conserved proline residues that are hydroxylated in the presence of oxygen [17,18]. This serves as a signal for the beta domain of pVHL to bind effectively to HIF-α, resulting in its polyubiquination and proteosomal degradation. In hypoxic or anoxic conditions, HIF-α does not get hydroxylated and is unable to bind pVHL and its levels remain elevated. Deletion mutations in VHL or point mutations in the elongin binding domain that result in loss of functional VHL result in high HIF-α levels [19]. Likewise, point mutations in the beta domain of pVHL cause a similar effect. VHL disease has been shown to display a complex genotype-phenotype correlation wherein specific VHL mutations are associated with a higher risk for a subset of VHL-associated tumor types [20-22]. The type 2A disease phenotype is marked by mutations in pVHL beta domain that hinder its ability to bind HIF-α. Interestingly, although these mutations lead to higher HIF-α levels, families with these mutations show a low incidence of renal cell carcinoma (RCC) [23]. This finding suggests the possibility of VHL activities other than HIF-α regulation, the loss of which leads to RCC.

Studies to date have shown that downregulation of HIF-α is necessary for VHL dependent tumor suppression in some model systems [24]. However, other model systems have shown that high levels of HIF-α alone are insufficient for malignant transformation [25]. Therefore, it is currently unclear what role HIF-α plays in VHL-associated tumors, especially with regard to initiation of renal carcinomas. In this report, we have examined the role of HIF-2α regulation in specific cellular phenotypes that have been previously associated with VHL. Notably, VHL replacement in VHL-null cells has been shown to regulate cellular morphology and organization, levels of cell surface integrins, fibronectin matrix assembly, and to direct growth arrest and cellular differentiation of cells grown on extracellular matrices [26-28]. Using an RNA interference approach to lower HIF-2α levels in VHL-null renal cell lines that express this HIF-α isoform only, we have investigated whether lower levels of HIF-2α can mimic the effects of VHL replacement in these cells. Here, we report that HIF-2α regulation plays a role in certain, but not all VHL-associated cellular phenotypes. These results suggest that pVHL has other important biochemical and cellular functions in addition to the ubiquitination of HIF-α that may be important for tumor suppression.

## Results

786-O and A498 VHL-null renal cell lines both contain high levels of HIF-2α, but not the other isoforms of HIF-α [16]. In order to investigate whether downregulation of HIF-2α is sufficient to mimic VHL replacement in these cells, we lowered the levels of HIF-2α using two retroviral vectors that direct the expression of short hairpin RNA (shRNA) directed against HIF-2α. These constructs are based on the pSuperRetro shRNA system [29] and have been previously shown to specifically inhibit HIF-2α in the 786-O cell line [24]. The empty vector, pSuperRetro, was used as a control.

**Figure 1:**
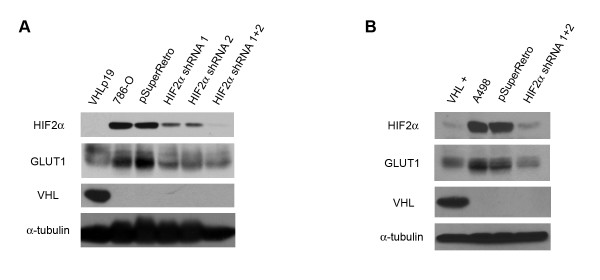
**Successful knockdown of HIF-2α in both 786-O and A498 renal cell lines**. (A) Lysates from 786-O cells stably transfected with VHL (VHL +), parental 786-O cells (786-O), and 786-O cells infected with an empty retrovirus (pSuperRetro) or with retroviruses encoding single HIF-2α shRNAs (HIF2α shRNA 1; HIF2α shRNA 2) or doubly infected with both shRNAs (HIF2α shRNA 1+2) were immunoblotted for HIF-2α (top panel), GLUT1 (upper middle panel) and VHL (lower middle panel). Bottom panel is an alpha tubulin immunoblot to demonstrate equal protein loading (25 μg) in each lane. (B) Lysates from A498 cells stably transfected with VHL (VHL +), parental A498 cells (A498), and A498 cells infected with an empty retrovirus (pSuperRetro) or doubly infected with both HIF-2α shRNAs (HIF2α shRNA 1+2) were immunoblotted as in (A).

We initially infected 786-O cells with single HIF-2α shRNA retroviruses and selected to obtain a pool of puromycin resistant cells. However, incomplete knockdown of HIF-2α levels was observed with either of the single shRNA constructs (Figure 1a, upper panel). To obtain a more robust downregulation of HIF-2α, 786-O cells were infected in succession with both HIF-2α shRNA-containing retroviruses. HIF-2α inhibition was observed in the doubly infected cells that mimicked the steady state levels seen in the presence of VHL under normoxia. In parallel, A498 cells were infected with the two shRNA retroviruses, resulting in efficient knockdown of HIF-2α (Figure 1b, upper panel). To ensure that the shRNA was not only diminishing levels of HIF-2α, but was also causing downregulation of HIF target genes, immunoblot analysis was performed for the GLUT1 protein. As anticipated, downregulation of GLUT1 coincided with downregulation of HIF-2α (Figure 1a, upper middle panel; Figure 1b, upper middle panel). Thus, in both 786-O and A498 cell lines, shRNA directed at HIF-2α lowered levels of both HIF-2α and a representative HIF-responsive target.

**Figure 2:**
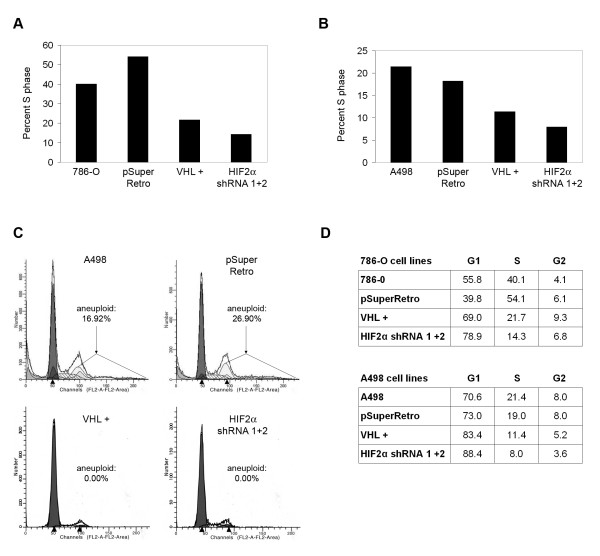
**Knockdown of HIF-2α mediates cell cycle arrest on collagen I**. (A) Parental 786-O cells (786-O), 786-O cells stably transfected with VHL (VHL +), and 786-O cells infected with an empty retrovirus (pSuperRetro) or doubly infected with both HIF-2α shRNAs (HIF2α shRNA 1+2) were grown for 2 weeks after attaining confluence on gels of polymerized collagen I. Flow cytometry was performed with a FACScan instrument. Percentages of cells in S phase are shown for each cell line. (B) FACS analysis was performed as in (A) using A498 cell lines. (C) Graphical representation of the FACS analysis in (B) with arrows highlighting cells with aneuploid DNA content. (D) Complete cell cycle profiles of 786-O and A498 cell lines (diploid cells only) from FACS analyses presented in (A), (B), and (C).

Downregulation of HIF-2α in 786-O and A498 cells has been shown to block tumor formation in nude mice [24,30]. One mechanism for this tumor suppression could involve cell cycle arrest. It has been previously shown that 786-O cells with replaced VHL arrest in the G_0_-G_1 _phase of the cell cycle when cultured on collagen I, whereas VHL negative cells do not [26]. Cell cycle arrest is not seen when these cells are cultured by standard methods (*i.e*., on plastic). To investigate whether HIF-2α plays a role in cell cycle arrest, 786-O and A498 cell lines were plated on collagen I and grown to high cell density (2 weeks post-confluency) and analyzed by FACS to assess cell cycle distributions. Note that under these conditions, the cell lines utilized formed multilayers of cells. Nonetheless, VHL replacement in 786-O cells led to a decreased percentage of cells in S-phase (Figures 2a and 2d) and an increased G1 population, as previously reported. Strikingly, knockdown of HIF-2α levels led to an even further decrease in the fraction of S-phase cells. Similar results were obtained using the A498 cell lines (Figures 2b and 2d). Of note, both parental and control-infected A498 cells contained a significant percentage of aneuploid cells, however A498 cells with VHL replaced and those with shRNA-lowered HIF-2α had no aneuploid cells (Figure 2c). On the whole, removal of HIF-2α appears to be sufficient to mediate cell cycle arrest and suppression of anueploidy in this experimental setting, suggesting that the *in vivo *tumor suppression observed may occur at least in part through this mechanism. However, while these findings indicate that HIF-2α removal is sufficient to block growth of VHL-associated renal tumors, it is not clear whether HIF-2α dysregulation alone can lead to the initiation of renal carcinomas.

**Figure 3:**
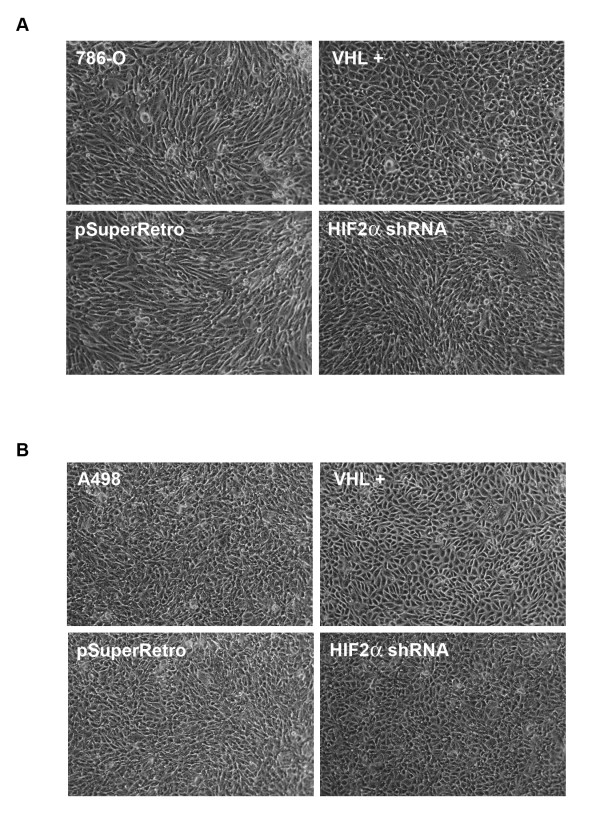
**Knockdown of HIF-2α has only a slight effect on cell morphology**. (A) Parental 786-O cells (786-O), 786-O cells stably transfected with VHL (VHL +), and 786-O cells infected with an empty retrovirus (pSuperRetro) or doubly infected with both HIF-2α shRNAs (HIF2α shRNA) were grown for 4 days on thin layers of collagen I. Images were captured by digital camera mounted on an inverted microscope. (B) Morphological analysis was performed as in (A) using A498 cell lines grown for 7 days on thin layers of collagen I.

In order to study the role of HIF-2α in processes that may be more relevant to the earlier stages of renal tumor development, we examined additional VHL-mediated activities. VHL has been shown to play a vital role in morphological differences of cells grown on collagen I gels, especially at high cell densities [26]. Under these conditions, 786-O cells are seen as disorganized, and branched with a fibroblastic spindly phenotype, whereas 786-O cells in which VHL is replaced form a monolayer of polygonal cells, with clear cell borders, characteristic of differentiated epithelia [26]. To determine if these cellular phenotypic differences are dependent on HIF-2α regulation, the 786-O and A498 cell lines were plated on collagen I and morphological characteristics were observed. As previously observed, VHL-negative parental and control-infected 786-O and A498 cells showed a disorganized fibroblastic, spindly phenotype, whereas cells with VHL replaced were seen as a monolayer of apparently more differentiated cells (Figure 3a and 3b). Also as previously observed, VHL-negative parental and control-infected cells displayed a spiral-shaped multicellular organization (which was more apparent with the 786-O lines) that may be related to augmented invasiveness and matrix remodeling properties and/or disrupted polarity of these cells [26,31-33]. Importantly, a slightly intermediate phenotype that more closely resembled VHL-negative cells was observed with both the 786-O and A498 cells containing shRNA-reduced levels of HIF-2α (Figure 3a and 3b).

**Figure 4:**
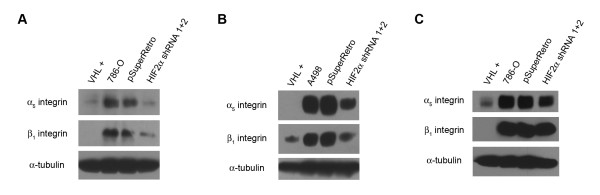
**Knockdown of HIF-2α has a partial effect on downregulation of α5 and β1 integrins**. (A) Lysates from 786-O cells stably transfected with VHL (VHL +), parental 786-O cells (786-O), and 786-O cells infected with an empty retrovirus (pSuperRetro) or doubly infected with both HIF-2α shRNAs (HIF2α shRNA 1+2) were immunoblotted for α5 integrin (top panel) and β1 integrin (middle panel). Bottom panel is an alpha tubulin immunoblot to demonstrate equal protein loading (25 μg) in each lane. (B) Integrin immunoblotting was performed as in (A) using A498 cell lines. (C) Integrin immunoblotting was performed as in (A), except cells were grown on thin layers of collagen I.

Cell morphology, motility, and cell spreading have been shown to be influenced by various integrin proteins that are expressed at the cell surface. It has been previously shown that levels of integrins β1, α3, and α5 are downregulated in 786-O cells with replaced VHL [26]. To determine the effect of HIF-2α levels on the expression of these integrins, we prepared lysates from our cell lines, grown for 7 to 14 days post-confluency on plastic, and subjected them to immunoblot analysis for α5 and β1 integrins (Figure 4a and 4b). Both 786-O and A498 cells with reduced HIF-2α levels demonstrated decreased levels of these integrins. However, the decreases in α5 integrin and especially β1 integrin were less striking than that seen with cells containing intact VHL, indicating that HIF-2α levels may play a partial role in this cellular phenotype. We also assessed the levels of α5 and β1 integrins for 786-O cells that were grown on thin layers of collagen I. Surprisingly, HIF-2α reduction had no effect on α5 and β1 integrin levels on cells grown on collagen I (Figure 4c), in stark contrast to VHL replacement, which efficiently decreased the levels of these integrins. Thus, proper cell signaling from extracellular matrices such as collagen I may require pVHL activities that are independent of or in addition to HIF-2α regulation.

**Figure 5:**
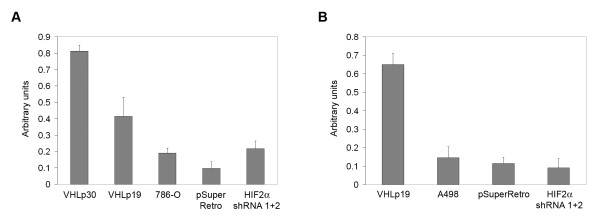
**Knockdown of HIF-2α has no effect on fibronectin deposition**. (A) The amount of fibronectin deposited by 786-O cells stably transfected with VHL (VHLp30 and VHLp19), parental 786-O cells (786-O), and 786-O cells infected with an empty retrovirus (pSuperRetro) or doubly infected with both HIF-2α shRNAs (HIF2α shRNA 1+2) was measured by ELISA at 405 nm. Error bars represent standard deviation. Differences between VHLp30 cells and all other cell lines were statistically significant (P < 0.03). (B) Fibronectin ELISA was performed as in (A) using A498 cells stably transfected with VHL (VHLp19), parental A498 cells (A498), and A498 cells infected with an empty retrovirus (pSuperRetro) or doubly infected with both HIF-2α shRNAs (HIF2α shRNA 1+2). Differences between VHLp19 cells and all other cell lines were statistically significant (P < 0.009).

It has been shown that cells that contain wild-type VHL properly assemble an extracellular fibronectin matrix [28,34]. A number of studies have suggested that this cellular VHL activity occurs independently of HIF-α regulation. In order to directly assess whether HIF-2α levels affect extracellular fibronectin matrix assembly, we quantified the amount of fibronectin deposited by our cell lines (Figure 5a and 5b). Both 786-O and A498 cells containing ectopically expressed VHL deposited higher concentrations of fibronectin in comparison to parental and control-infected cells. However, 786-O and A498 cells with shRNA-reduced HIF-2α did not show increased fibronectin deposition as compared to control or parental cells and were significantly deficient in comparison to cells with VHL (P < 0.006 with 786-O VHLp30 cells and P < 0.009 with A498 VHLp19 cells, Student's paired, two tailed t-test), indicating that VHL-mediated fibronectin deposition occurs independently of HIF-2α regulation. It should be noted that in these assays, 786-O cells expressing VHLp19 deposited significantly lower amounts of fibronectin than those expressing VHLp30 (P < 0.03, Student's t-test) and although the levels for either of the VHL-expressing cells were greater than the parental and control 786-O cells, differences between VHLp19 cells and parental or control cells did not achieve statistical significance at the 95% confidence interval (P > 0.05 and P > 0.06, respectively, Student's t-test). However, fibronectin deposition from A498 cells expressing VHLp19 was significantly greater than A498 parental and control cells (P < 0.005 and P < 0.004, respectively), indicating that VHLp19 does contain this function and that the differences seen in fibronectin deposition among the VHL-expressing 786-O cells may be clonal in origin since these cell lines were derived from single-cell clones [3]. Regardless of these minor differences, knockdown of HIF-2α did not increase fibronectin deposition in either cell line, in contrast to the effect seen upon VHL replacement.

**Figure 6:**
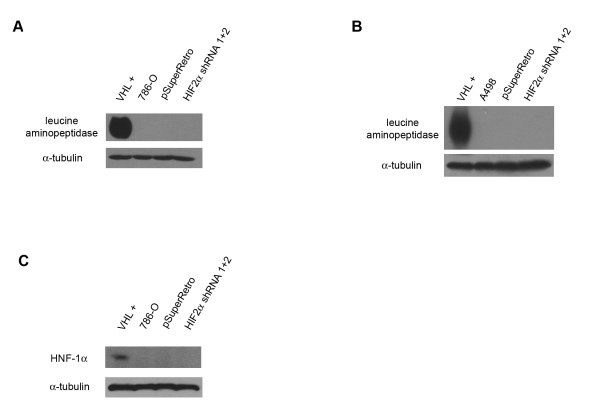
**Knockdown of HIF-2α does not cause renal cell differentiation**. (A) Lysates from 786-O cells stably transfected with VHL (VHL +), parental 786-O cells (786-O), and 786-O cells infected with an empty retrovirus (pSuperRetro) or doubly infected with both HIF-2α shRNAs (HIF-2α shRNA 1+2) were immunoblotted for the renal marker leucine aminopeptidase (top panel). Bottom panel is an alpha tubulin immunoblot to demonstrate equal protein loading (25 μg) in each lane. (B) Leucine aminopeptidase immunoblotting was performed as in (A) using A498 cells lines. (C) HNF-1α immunoblotting was performed on 786-O cell lines, as in (A).

VHL expression has been previously shown to direct the expression of cell surface markers of renal cell differentiation in 786-O cells [26]. Prior studies have shown that pVHL-mediated differentiation of 786-O renal cells is dependent on high cell density [26]. Accordingly, 786-O and A498 cell lines were grown for fourteen days post-confluency on plastic tissue culture dishes to allow for differentiation, and immunoblot analyses were performed using antibodies to the renal cell surface marker leucine aminopeptidase, a brush border enzyme found in proximal tubule epithelium [35]. Expression of leucine aminopeptidase was detected in both the 786-O and A498 cells containing ectopically expressed VHL (Figure 6a and 6b). As expected, expression of this differentiation marker was not detected in the parental or control cell lines. Notably, HIF-2α shRNA cell lines also showed no detectable expression of leucine aminopeptidase. Expression of the renal marker HNF-1α was also observed in 786-O cells expressing pVHL, but not in cells with reduced HIF-2α levels (Figure 6c). These results indicate that HIF-2α regulation alone does not cause renal cell differentiation and that the differentiation process in renal cells is dependent on other VHL activities.

## Discussion

VHL has been linked to a number of phenotypes at the cellular level that may be important for tumor suppression. In this study, we have analyzed whether certain cellular VHL functions are dependent on pVHL's ability to regulate HIF-2α. We chose cell lines lacking HIF-1α to specifically examine the isolated role of HIF-2α, and used retroviral infection to obtain multiclonal pools, limiting the potential of specific clonal populations. We report that HIF-2α downregulation was sufficient to cause cell cycle arrest, whereas VHL-associated cellular morphological changes and downregulation of α5 and β1 integrins were minimally dependent on HIF-2α regulation. Notably, HIF-2α knockdown was not sufficient to bring about proper fibronectin matrix assembly and differentiation of renal cells, both of which are normally mediated by VHL.

Downregulation of HIF-2α levels by shRNA has been shown to be sufficient to suppress tumor growth of VHL-defective renal carcinoma cells in nude mice [24,30]. In the present study, decreased levels of HIF-2α led to cell cycle arrest of cells grown on collagen I, suggesting a mechanism for the *in vivo *growth arrest. Additionally, knockdown of HIF-2α abrogated aneuploidy in the A498 cell line. These findings reinforce the notion that HIF-2α is important in the progression of VHL-associated tumors. This role in renal cells may be specific to HIF-2α (rather than HIF-1α), since HIF-2α preferentially transactivates protumorigenic genes, including cyclin D1 and vascular endothelial growth factor (VEGF) [36]. However, while many of the clinical manifestations of VHL inactivation, including tumors with a high degree of vascularization, might be explained by upregulated hypoxia-inducible genes, it is doubtful whether HIF-α dysregulation (or HIF-2α dysregulation) alone can initiate renal tumorigenesis. In support of this notion, common mutations in the HIF-α binding domain of pVHL that fall within the type 2A disease phenotype result in higher levels of HIF-α, yet are associated with a low incidence of RCC [23]. Additionally, constitutive expression HIF-2α was seen to be less invasive in Matrigel than loss of VHL [37]. Overexpression of HIF-2α was also seen to reduce the growth of rat glioma tumors and cause apoptosis [38]. Collectively, these findings suggest that HIF-2α overexpression contributes to tumorigenesis, but is insufficient for the initiation of renal carcinomas. Thus, loss of some other VHL-associated function is required for initiation of renal carcinomas.

Recent studies have focused on a VHL role in earlier stages of renal tumor development, namely renal cyst formation [33,39,40]. These studies have suggested that renal cyst formation may rely more on disruption of VHL functions such as cell polarity and cilia formation, although the importance of HIF-α regulation for these phenomena was not in agreement [33,39]. The role of HIF-2α on other aspects of cellular morphology is also currently unclear. In the present studies, HIF-2α knockdown had only a modest effect on cell morphology and on expression of α5 and β1 integrins. In other studies assessing various parameters of cell morphology and cell-to-cell interaction, overexpression of a constitutively active HIF-2α was seen to have no effect on adherens junctions (as assayed by β-catenin) and tight junction organization [32]. On the other hand, HIF-2α has been seen to regulate the expression of another adherens junction protein, E-cadherin [39,41]. A more precise determination of the contribution of HIF-2α toward adherens junction activities as well the exact relationship of these markers to VHL-mediated renal tumorigenesis would clarify these findings.

Fibronectin matrix assembly has been suggested to be a key VHL function for VHL tumor suppression [37]. In the present studies, fibronectin deposition was shown to be unaffected by HIF-2α knockdown. Similarly, cells with constitutive expression of HIF-2α have been shown to have intact assembly of fibronectin and collagen IV extracellular matrices [37] and conversely, a pVHL mutant that retained its ability to ubiquitinate HIF-α failed to promote fibronectin matrix assembly [34]. These findings all support the notion that this VHL function is an HIF-α independent phenotype. However, it is unlikely that loss of normal fibronectin assembly is responsible for the initiation of renal tumors, since VHL proteins harboring type 2C disease mutations are defective in fibronectin assembly, yet these mutations are associated with a low risk of RCC. Also, the differences observed in fibronectin deposition between 786-O cell lines expressing either of the two isoforms of VHL in the present studies might indicate a more subtle role of fibronectin, since these cell lines have been shown to have equal tumor suppression ability [3].

Another non-HIF-2α associated phenotype observed in the present studies was VHL-dependent expression of renal differentiation markers. It is likely that dedifferentiation or failure to differentiate is a very early step in renal tumorigenesis. Notably, dedifferentiation has been observed in renal cysts following inactivation of VHL and changes in a number of renal cell differentiation markers have been observed in renal cysts [40,42]. Differentiation may also underlie the formation of primary cilium that is seen upon VHL replacement in VHL-null cells [33,39]. Interestingly, a type 2A VHL mutant (low risk of RCC) was seen to support ciliogenesis [33], suggesting that with type 2A VHL mutations, renal cells remain differentiated and are therefore not capable of initiating a tumor. In contrast to type 2A VHL disease, specific mutants within the elongin binding site (type 2B phenotype) correlate with a high incidence of renal cell carcinoma [23]. Since these mutations disrupt VHL-mediated ubiquitination as a whole, it is possible that there is another substrate that the pVHL complex targets, which is dysregulated in type 2B, but not type 2A VHL disease. This alternative substrate of pVHL may be more directly linked to cell differentiation than HIF-2α and may be more relevant to initiation of renal carcinomas. However, non-ubiquitination functions of VHL, which have yet to be fully elucidated, may prove to be more crucial for VHL-mediated differentiation. In either case, it is likely that the loss of VHL-mediated differentiation synergizes with the effects of high HIF-α levels to lead to the development of renal carcinomas.

## Conclusion

HIF-2α regulation plays a role in certain, but not all VHL-associated cellular phenotypes. Notably, HIF-2α regulation is insufficient for pVHL-associated renal cell differentiation. These results suggest that pVHL has other important biochemical and cellular functions in addition to the ubiquitination of HIF-α that may be important for tumor suppression.

## Methods

### Cell lines and cell culture

All renal cell lines (293T, 786-O and A498) were obtained from the American Type Culture Collection. All cells were grown in Dulbecco's modified Eagle's medium containing 10% Serum Supreme (BioWhitaker) and penicillin-streptomycin (100 U/ml and 10 μg/ml, respectively). The 786-O and A498 renal carcinoma cell lines both contain a single VHL allele harboring a frameshift mutation (at codons 104 and 142, respectively) [1,43]. 786-O cells stably transfected with VHLp19 and VHLp30 have been described previously [3]. A498 cells stably transfected with VHLp19 plasmid [3] were isolated following selection with media containing 600 μg/ml of G418 [33]. To condition cells at high density, cells were allowed to grow for 1–2 weeks post-confluency with media replenishment every 48 h. For culture on collagen I, cells were either grown on purchased plates coated with a thin layer of collagen I (Becton Dickinson) or on an approximate 2–3 mm thick layer of rat collagen I (Becton Dickinson), prepared as previously described [26]. FACS analysis of cells grown 2 weeks post-confluency on thick layers of collagen I was performed as previously described [26]. Analysis of DNA histograms was performed using ModFitLT 3.1 software (Verity Software House). The first major G1 peak with a corresponding G2 peak (containing DNA content double that of G1) was classified as the diploid cell population, whereas cell populations displaying alternative G1 peaks with corresponding G2 peaks were classified as anueploid.

### Plasmids, Retroviral Production, and Infection

Two retroviral plasmids directing expression of short hairpin RNA's (shRNA) containing unique HIF-2α 19mer sequences [24] were generously provided by Dr. William Kaelin (Dana Farber Cancer Center). To produce retroviral supernatants, each retroviral plasmid was transfected into 293T cells plated in duplicate on 35 mm dishes using Lipofectamine Plus reagent (Invitrogen) as directed by the manufacturer. Each transfection mix also received the retroviral packing plasmid, pCL-Ampho [44]. Following transfection, 293T cells from duplicate transfections were pooled and replated into 100 ml dishes. Medium containing retroviruses was collected at 72 h post-transfection and filtered through a 45 μm pore-size filter. To create stable pools of retrovirally infected cells, 786-O and A498 cells were incubated overnight in a mixture (1:1) of retroviral supernatant and fresh medium supplemented with polybrene (10 μg/ml). Two days later, cells were selected with puromycin (1 μg/ml) for 10 to 14 days.

### Immunoblotting

For all immunoblots, cells were grown to 100% confluency. Culture plates were rinsed with phosphate buffer saline (PBS) and cells were lysed using 500 μl of lysis buffer (50 mM HEPES (pH 7.6), 250 mM NaCl, 0.1% Nonidet P-40, 5 mM EDTA, 1 mM phenylmethylsulfonyl fluoride (PMSF), 1 mM Na_2_VO_3 _and 2 μg/ml each of aprotinin, bestatin, and leupeptin), incubating at 4°C for 30 minutes. To prepare lysates of cells grown at high density, the lysis buffer was supplemented with 0.5% Triton X-100 and 0.5% Nonidet P-40 to ensure solubilization of proteins. Lysed cells were scraped with a plastic scraper, resuspended by pipetting, collected and then microcentrifuged for 15 minutes. Supernatant containing clarified protein lysate was removed and protein levels were determined by Bradford assay (Bio-Rad). Equal amounts (25 μg) of protein lysates were added to an appropriate amount of 3.5× SDS PAGE loading buffer (175 mM Tris (pH 6.8), 0.75 mM DTT, 7% SDS, 35% glycerol, 0.35% bromophenol blue) and resolved by SDS-polyacrylamide gel electrophoresis and subsequently transferred to a polyvinylidene difluoride (PVDF) membrane overnight. Rabbit polyclonal anti-HIF-2α antibody was from Novus Biological. Rabbit polyclonal anti-Glut-1 antibody was from Alpha Diagnostics. Anti-VHL mAb 11E12 has been previously described [3]. Anti-leucine aminopeptidase mAb was obtained from Lab Vision/NeoMarkers. Integrin and HNF-1α mAbs were obtained from BD Transduction Laboratories. Rabbit anti-fibronectin antibody, anti-alpha tubulin mAb, as well as all secondary rabbit and mouse antibodies used were from Sigma.

### Fibronectin ELISA

Cells (2 × 10^4^) were plated in triplicate in a 96-well tissue culture plate and grown for six days. Cell removal was performed with Phosphate Buffered Saline (PBS) containing 2 mM EDTA, incubating at 37°C for 5–10 minutes. Complete removal of cells was confirmed by microscopy. Plates were blocked using TBS-Tween/0.1% BSA (Tris buffered saline containing 0.5% Tween-20 and 0.1% heat-inactivated Bovine Serum Albumin) for 45 minutes at 37°C and subsequently incubated with 2 μg/ml of rabbit anti-fibronectin antibody in TBS-Tween/0.1% BSA for one hour. Plates were washed three times with TBS-Tween and then incubated for 45 minutes at 37°C with anti-rabbit secondary antibody, diluted to a final concentration of 0.1 μg/ml with TBS-Tween/0.1% BSA. Plates were again washed three times with TBS-Tween and color was developed with 200 μl of p-nitrophenyl phosphate (Sigma) in ELISA substrate buffer (1 mg/ml in 50 mM NaCO_3_, 1 mM MgCl_2_, pH 9.8). Reactions were stopped with 50 μl of 3 M NaOH, and the OD at a wavelength of 405 nm was determined for each well in a microplate reader (BioRad).

## Abbreviations

Hypoxia-inducible factor (HIF), renal cell carcinoma (RCC), short hairpin RNA (shRNA), von Hippel-Lindau (VHL).

## Competing interests

The author(s) declare that they have no competing interests.

## Authors' contributions

MDH and EK were involved in the overall study design and coordination and performed most of the experimental procedures and data analyses. AEA performed some of the cell culture and western blot analyses. MDH wrote the original draft of the manuscript, and RDB and ARS edited the manuscript. RDB contributed to the study design. ARS conceived of the study and served as the principal investigator.
